# Comparison among conventional and advanced MRI, ^18^F-FDG PET/CT, phenotype and genotype in glioblastoma

**DOI:** 10.18632/oncotarget.21482

**Published:** 2017-10-04

**Authors:** Maria Consuelo Valentini, Marta Mellai, Laura Annovazzi, Antonio Melcarne, Tetyana Denysenko, Paola Cassoni, Cristina Casalone, Cristiana Maurella, Silvia Grifoni, Piercarlo Fania, Angelina Cistaro, Davide Schiffer

**Affiliations:** ^1^ Department of Neuroradiology, A.O.U. Città della Salute e della Scienza, 10126 Turin, Italy; ^2^ Research Center/Policlinico di Monza Foundation, 13100 Vercelli, Italy; ^3^ Department of Neurosurgery, A.O.U. Città della Salute e della Scienza, 10126 Turin, Italy; ^4^ Department of Medical Sciences, University of Turin, 10126 Turin, Italy; ^5^ Istituto Zooprofilattico Sperimentale del Piemonte, Liguria e Valle d’Aosta, 10154 Turin, Italy; ^6^ Positron Emission Tomography Center IRMET S.p.A, Euromedic Inc., 10136 Turin, Italy; ^7^ Institute of Cognitive Sciences and Technologies, National Research Council, 00185 Rome, Italy

**Keywords:** glioblastoma, MRI, ^18^F-FDG PET/CT, phenotype, genotype

## Abstract

Glioblastoma (GB) is a highly heterogeneous tumor. In order to identify *in vivo* the most malignant tumor areas, the extent of tumor infiltration and the sites giving origin to GB stem cells (GSCs), we combined positron emission tomography/computed tomography (PET/CT) and conventional and advanced magnetic resonance imaging (MRI) with histology, immunohistochemistry and molecular genetics. Prior to dura opening and tumor resection, forty-eight biopsy specimens [23 of contrast-enhancing (CE) and 25 of non-contrast enhancing (NE) regions] from 12 GB patients were obtained by a frameless image-guided stereotactic biopsy technique.

The highest values of 2-[18F]-fluoro-2-deoxy-D-glucose maximum standardized uptake value (^18^F-FDG SUV_max_), relative cerebral blood volume (rCBV), Choline/Creatine (Cho/Cr), Choline/N-acetylaspartate (Cho/NAA) and Lipids/Lactate (LL) ratio have been observed in the CE region. They corresponded to the most malignant tumor phenotype, to the greatest molecular spectrum and stem cell potential. On the contrary, apparent diffusion coefficient (ADC) and fractional anisotropy (FA) in the CE region were very variable.

^18^F-FDG SUV_max_, Cho/Cr and Cho/NAA ratio resulted the most suitable parameters to detect tumor infiltration. In edematous areas, reactive astrocytes and microglia/macrophages were influencing variables.

Combined MRI and ^18^F-FDG PET/CT allowed to recognize the specific biological significance of the different identified areas of GB.

## INTRODUCTION

Glioblastoma (GB), the most malignant and frequent glioma, is a phenotypically and genotypically heterogeneous tumor; for this region, one single region of the tumor cannot be considered as representative for the entire neoplasia [1]. GB is characterized by progressive events (cell proliferation, increased vessel density and angiogenesis, diffusion, infiltration) and by regressive events (necrosis, hemorrhages, vascular thrombosis). Infiltration is an important issue in the tumor control; it is not uniform along the tumor borders and, often, it is so mild to be practically undetectable. Tumor cells can migrate through the normally looking *corpus callosum* and isolated tumor cells (ITCs) can be found very far from tumor borders [2].

Tumor heterogeneity can be detected combining conventional (standard T1- and T2-weighted sequences) and advanced magnetic resonance imaging (MRI), including perfusion-weighted imaging (PWI), diffusion-weighted imaging (DWI), diffusion-tensor imaging (DTI) and MR spectroscopic imaging (MRSI) [3, 4], with positron emission tomography (PET)/computed tomography (PET/CT) [5].

Advanced MRI allows the identification of the tissue composition by multiple techniques. PWI directly measures the tissue microvasculature and it may be used to evaluate tumor neoangiogenesis and malignancy grade. The measurement of changes in the signal intensity caused by the intravascular bolus of paramagnetic contrast agents results in perfusion parameters [cerebral blood volume (CBV), mean transit time (MTT), cerebral blood flow (CBF), peak height (PH) and percentage of signal intensity recovery (PSR)]. Relative CBV (rCBV) is the most reliable parameter for tumor microvasculature and, therefore, the most extensively used in tumor diagnosis [6–9]. Information on angiogenesis from rCBV maps positively correlates with the histopathological findings of increased tumor vascularity [10–13].

DWI, expressed by the apparent diffusion coefficient (ADC), measures the water molecule diffusion in biological tissues quantifying the proton Brownian movement. The intra-tumor diffusivity, due to the presence of different tumor components, is generally heterogeneous, reduced in areas of high cell density and increased in necrotic ones. ADC would be expected to be inversely correlated with glioma grade [9, 14, 15]. However, its clinical significance is limited by the glioma tissue heterogeneity [16] and the ADC value overlap [17].

DTI and the resulting parameters, as fractional anisotropy (FA) and mean diffusivity (MD), are microstructural quantitative measurements of the differences in anisotropic diffusion within the white matter tracts. Previous studies proved the role of DTI in characterizing tumor and peritumor involvement of white matter fibers [18].

In MRSI, various metabolites, such as Choline (Cho), Creatine (Cr), N-acetylaspartate (NAA), Myo-inositol (MI), Lipids and Lactate (LL) are reliable indicators of the histological grade. In particular, an increase of Cho with reduction of NAA and consequent variations of Cho/Cr and NAA/Cho ratio, significantly correlates with tumor grade and malignancy. Increased Cho values positively correspond to the Ki-67/MIB-1 labeling index (LI) used to evaluate the proliferative cellular activity [9].

Imaging with 2-[18F]-fluoro-2-deoxy-D-glucose (^18^F-FDG) was the first oncologic application of PET in human brain tumors [19]. ^18^F-FDG is actively transported across the blood brain barrier into the cells where it is phosphorylated [20]. ^18^F-FDG uptake is generally elevated in high grade tumors [21, 22] and can be evaluated by several parameters including the maximum standardized uptake value (SUV_max_) or the local metabolic rate of glucose. At present, ^18^F-FDG SUV_max_is the most frequently used parameter in clinical practice and its quantification is considered as predictive [23]. Increased intra-tumor glucose consumption directly correlates with tumor grade, cell density, biological aggressiveness and patient survival in both primary and recurrent gliomas [24]. Nevertheless, the diagnostic application of ^18^F-FDG PET/CT may have some limitations, due to the high rate of physiologic glucose metabolism in normal brain tissue. Co-registration of ^18^F-FDG PET/CT with MR images greatly improves its performance and the accuracy of interpretation. Reference to the MR image delineates the area of interest.

From the therapeutic point of view, the correlation of neuroimaging with tumor phenotype and genotype provides information on the biological significance of different tumor areas [25].

In this study we compared contrast enhancing (CE) and non-contrast enhancing (NE) tumor areas detected by conventional and advanced MRI (PWI, DWI, DTI and MRSI) and ^18^F-FDG PET/CT with histology, immunohistochemistry, molecular genetics and stemness potential in 48 biopsy specimens from 12 adult GB patients. Biopsies were removed by a frameless image-guided stereotactic system before craniotomy thus minimizing the loss of cerebrospinal fluid and brain displacement.

The aim of the study was the *in vivo* identification of (i) the most malignant tumor areas, (ii) the extent of tumor infiltration and (iii) the tumor sites where GB stem cells (GSCs) can be detected.

## RESULTS

A total of 48 biopsy specimens was collected from 12 adult GB patients (average four *per* case). Twenty-three samples were obtained from CE regions, 7 of which with a necrotic component, 13 and 12 from NE regions < 1 cm and > 1 cm, respectively.

### MRI and ^18^F-FDG PET/CT parameters according to the distance from the CE region

MRI parameters and ^18^F-FDG SUV_max_ variations in CE and NE regions according to their distance from the CE region are illustrated in Table 1.

**Table 1:** Comparison of MRI parameters and 18F-FDG SUV_max_ in CE and NE regions

Distance from the CE region	rT1C median/range	rT2 FSE median/range	rT2 FLAIR median/range	rCBV median/range	ADC median/range	FA median/range	MD median/range	Cho/Cr median/range	Cho/NAA median/range	NAA/Cr median/range	LL median/range	^18^F-FDG SUV_max_ median/range
CE tumor (n = 23)	1.90 (0.74-2.40)	1.51 (1.07-2.93)	1.58 (1.12-2.75)	4.16 (1.87-8.19)	1.26 (0.78-1.27)	0.189 (0.104-0.282)	1 (0.94-2.0)	2.70 (1.26-4.76)	1.95 (0.924-3.88)	0.906 (0.664-2.77)	5767 (3276-30780)	7.58 (3.93-10.99)
NE < 1 cm (n = 13)	1.115 (0.92-1.60)	1.185 (0.92-1.72)	1.775 (0.78-2.50)	2.415 (0.89-4.39)	1.26 (0.833-1.85)	0.286 (0.099-0.333)	1 (0.87-2.0)	1.86 (0.971-2.24)	1.835 (0.748-4.04)	1.135 (0.16-1.92)	3212 (2044-13140)	3.5 (2.36-6.42)
NE > 1 cm (n = 12)	0.92 (0.80-1.55)	1.55 (0.96-3.34)	2.46 (0.93-2.90)	1.23 (0.60-2.40)	1.60 (0.78-1.90)	0.28 (0.101-0.465)	1 (0.08-2.0)	1.47 (0.955-2.99)	1.08 (0.74-1.85)	1.27 (0.822-2.70)	3605 (3359-18900)	2.98 (2.04-6.01)

MRI, magnetic resonance imaging; ^18^F-FDG SUV_max_, 2-[18F]-fluoro-2-deoxy-D-glucose target tumor maximum standardized uptake value; CE, contrast enhancing; NE, non-contrast enhancing; rT1C, relative T1-weighted contrast enhancement; rT2 FSE, relative T2-weighted fast spin echo; rT2 FLAIR, relative T2-weighted fluid attenuated inversion recovery; rCBV, relative cerebral blood volume; ADC, apparent diffusion coefficient (10^-3^ mm^2^/s); FA, fractional anisotropy; MD, mean diffusivity (10^-3^ mm^2^/s); Cho/Cr, choline/creatine; Cho/NNA, choline/N-acetyl aspartate; NAA/Cr, N-acetyl aspartate/creatine; LL, lipids/lactate.

Correlation values were calculated by measurements from all 48 biopsy specimens. They are expressed as median and range values.

**Figure 1:**
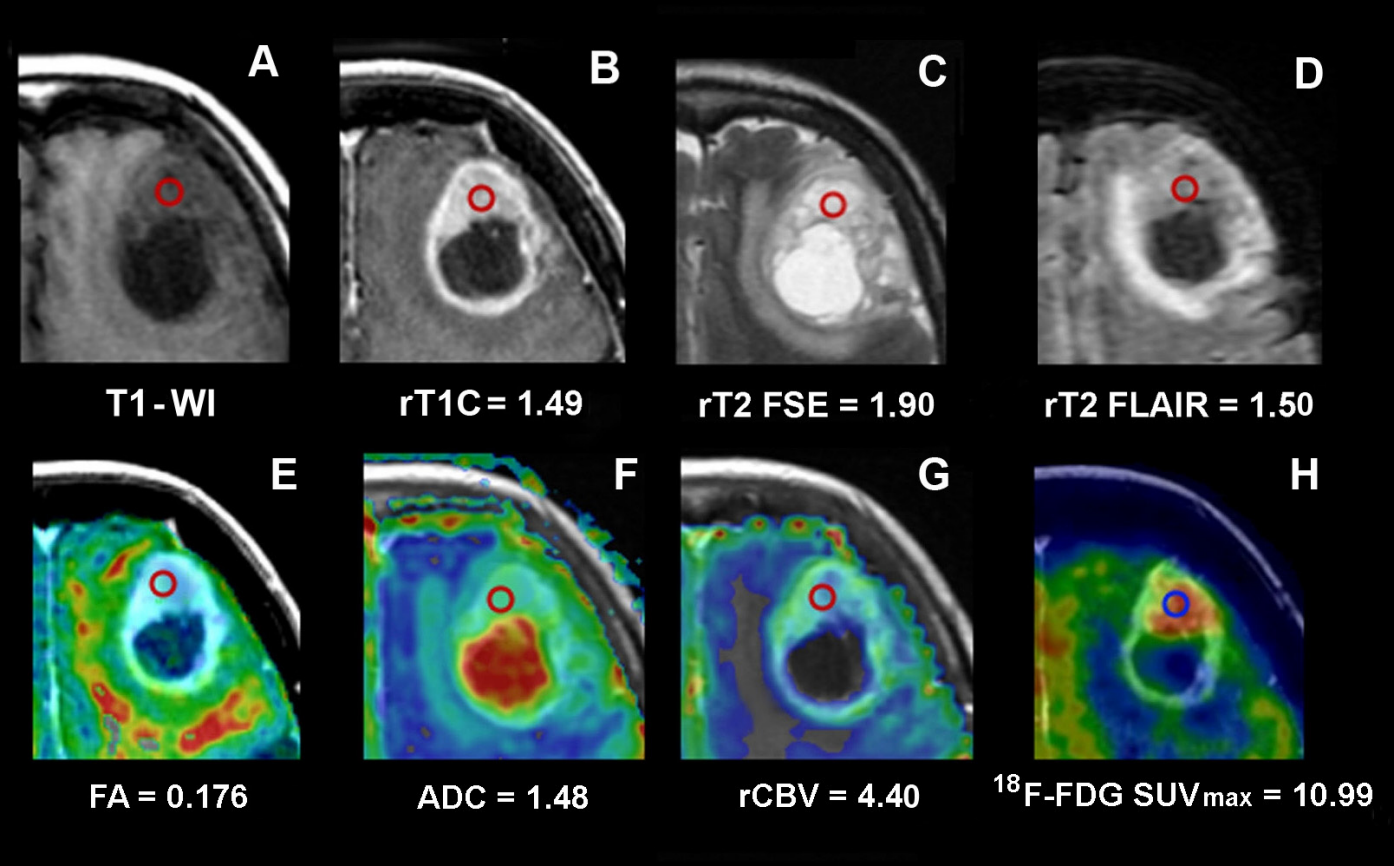
Conventional, advanced MRI and ^18^F-FDG PET/CT/MR fusion image of a frontal GB Images of a frontal GB in a 77-year-old woman (Case CTO3) and values from the ROI in the CE. The ROI is indicated by the red circle. **(A)** T1 weighted MRI. **(B)** T1C MRI and rT1C value. **(C)** T2 FSE MRI and rT2 FSE value. **(D)** T2 FLAIR MRI and rT2 FLAIR value. **(E)** FA color map fused with axial T1C MRI and FA value. **(F)** ADC color map fused with axial T1C MRI and ADC value. **(G)** rCBV color map fused with axial T1C MRI and rCBV value. **(H)** ^18^F-FDG PET/CT/MR fusion image and ^18^F-FDG SUV_max_ value. MRI, magnetic resonance imaging;^18^F-FDG PET/CT/MR, 2-[18F]-fluoro-2-deoxy-D-glucose positron emission tomography/computed tomography/magnetic resonance; CE, contrast enhancement; GB, glioblastoma; ROI, region of interest; T1-WI, T1-weighted image; T1C, T1-weighted contrast enhancement; T2 FSE, T2-weighted fast spin echo; T2 FLAIR, T2-weighted fluid attenuated inversion recovery; FA, fractional anisotropy; ADC, apparent diffusion coefficient; rCBV, relative cerebral blood volume; SUV_max_, maximum standardized uptake value.

The CE region showed the highest values of rT1C, rCBV, Cho/Cr, Cho/NAA and LL metabolite ratio, and of ^18^F-FDG SUV_max_ in comparison with NE regions (Figure 1 and Table 1). rT2 FSE, rT2 FLAIR and ADC were variable and did not show a clear-cut difference between CE and NE regions. Cho/Cr and Cho/NAA ratio decreased proportionally with ^18^F-FDG SUV_max_ whereas NAA/Cr ratio increased.

Both rCBV and FA significantly varied according to the distance from the CE region. In particular, rCBV values decreased in NE areas both < 1 cm (*β* = -2.6, *p* = 0.0001) and > 1 cm from the CE region (*β* = -1.556, *p* = 0.0001). In this latter, FA values were higher in non-necrotic samples. They significantly increased from CE to NE regions, with statistical significance in NE < 1 cm areas (*β* = 0.072, *p* = 0.037).

^18^F-FDG SUV_max_ proportionally decreased with the distance from the CE region (*β* = -2.581, *p* = 0.0001 for NE areas < 1 cm and *β* = - 3.174, *p* = 0.0001 for NE areas > 1 cm, respectively).

Among all MRI parameters, rCBV (*β* = 1.50, *p* = 0.039), Cho/Cr ratio (*β* = 2.048, *p* = 0.003) and rT1C (*β* = 1.342, *p* = 0.037) were significantly associated with ^18^F-FDG SUV_max_ in the CE region and also regardless of the distance from the CE region (*p* < 0.05 for all).

### Comparison of regional MRI and ^18^F-FDG PET/CT parameters with histopathological findings

The histological features identified in the biopsy specimens resulted in four patterns. Pattern 1 (P-1) corresponded to the CE region characterized by the highest values of cell and vessel density, nuclear polymorphism, Ki-67/MIB-1 LI, circumscribed necroses and endothelial hyperplasia; pattern 2 (P-2) was characterized by highly infiltrated tissue with satellitosis or tumor cell co-option with neoangiogenesis or microvascular proliferations (MVPs); pattern 3 (P-3) was characterized by a mild peripheral tumor infiltration without neoangiogenesis and MVPs; pattern 4 (P-4) was characterized by tumor infiltration with peritumor edema. The number of biopsy specimens displaying the above mentioned patterns was uneven: however, at least P-1 to P-3 were always present, while P-4 variably occurred.

**Figure 2:**
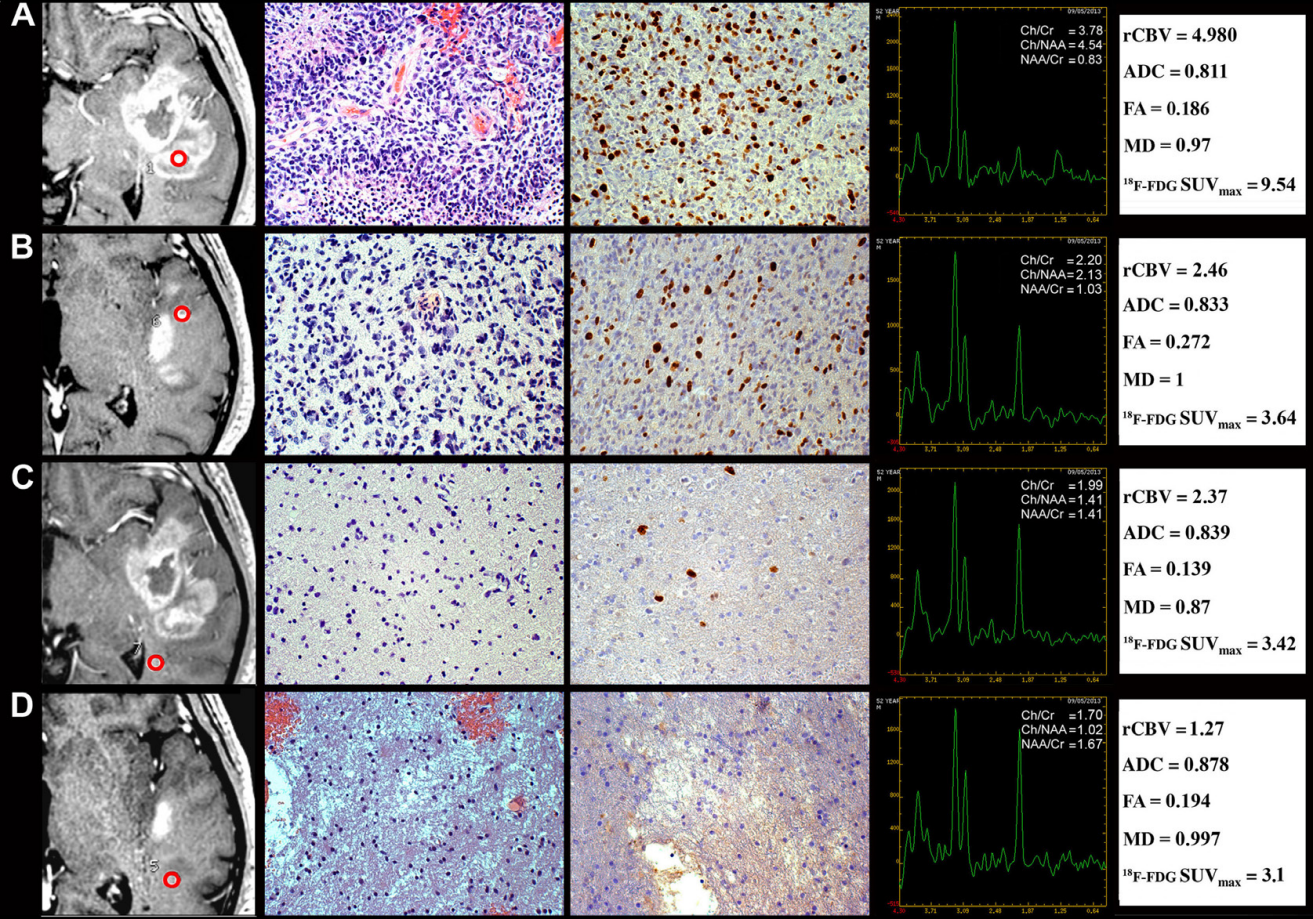
Regional correlation of MRI/MRSI and ^18^F-FDG PET/CT parameters from CE and NE regions with histopathology of a GB stereotactic biopsy specimen A 52-year-old man with temporal GB (Case CTO11). **(A)** CE biopsy specimen corresponding to pattern 1. Column 1, T1C MRI (the ROI is indicated by the red circle). Column 2, Hyperproliferative tumor area (H&E, 200×). Column 3, Ki-67/MIB-1 LI (DAB, 200×). Column 4, MRSI. Column 5, rCBV, ADC, FA, MD and ^18^F-FDG SUV_max_values. **(B)** NE < 1 cm biopsy specimen corresponding to pattern 2. Column 1, T1C MRI. Column 2, high tumor infiltration area (H&E, 200×). Column 3, Ki-67/MIB-1 LI (DAB, 200×). Column 4, MRSI. Column 5, rCBV, ADC, FA, MD and ^18^F-FDG SUV_max_ values. **(C)** NE > 1 cm biopsy specimen corresponding to pattern 3. Column 1, T1C MRI. Column 2, mild tumor infiltration area (H&E, 200×). Column 3, Ki-67/MIB-1 LI (DAB, 200×). Column 4, MRSI. Column 5, rCBV, ADC, FA, MD and^18^F-FDG SUV_max_ values. **(D)** NE > 1 cm biopsy specimen corresponding to pattern 4. Column 1, T1C MRI. Column 2, tumor infiltration area with edema (H&E, 200×). Column 3, Ki-67/MIB-1 LI (DAB, 200×). Column 4, MRSI. Column 5, rCBV, ADC, FA, MD and ^18^F-FDG SUV_max_ values. MRI, magnetic resonance imaging; MRSI, magnetic resonance spectroscopic imaging; ^18^F-FDG PET/CT, 2-[18F]-fluoro-2-deoxy-D-glucose positron emission tomography/computed tomography; CE, contrast enhancement; NE, non-contrast enhancement; GB, glioblastoma; ROI, region of interest; T1C, T1-weighted contrast enhancement; H&E, haematoxylin and eosin; LI, labeling index; DAB, 3,3'-Diaminobenzidine; SUV_max_, maximum standardized uptake value.

The comparison of the histological patterns with regional MRI parameters and ^18^F-FDG SUV_max_ is illustrated in Table 2. They were expressed as median and range of values for each pattern. The CE region represented the maximum of the tumor histological malignancy (P-1) and showed the maximum values of rCBV, Cho/Cr, Cho/NAA, LL and ^18^F-FDG SUV_max_ (Figure 2A and Table 2). rCBV values significantly decreased from P-1 to P-4 (*p* < 0.05 for all). Moreover, rCBV showed higher values in specimens from the CE region without macroscopic necrosis than in those containing necrosis. In presence of MVPs, rCBV remained high, also in areas far from the CE region.

**Table 2:** Comparison of MRI parameters and 18F-FDG SUV_max_ with the different histological patterns

Histological pattern	rCBV median/range	ADC median/range	FA median/range	MD median/range	Cho/Cr median/range	Cho/NAA median/range	NAA/Cr median/range	LL median/range	^18^F-FDG SUV_max_ median/range
P-1: CE tumor (n = 16)	4.325 (2.06-5.60)	1.265 (0.78-1.60)	0.186 (0.104-0.282)	1.0 (0.94-2)	2.465 (1.26-4.76)	1.94 (0.924-3.88)	1.14 (0.904-2.77)	6789 (3276-30780)	8.18 (3.93-10.99)
P-2: high infiltration with MVPs (n = 10)	2.78 (1.23-4.69)	1.39 (0.83-1.85)	0.241 (0.104-0.465)	1.0 (1-2)	2.24 (1.72-4.76)	1.87 (1.08-5.40)	1.04 (0.37-2.70)	3359 (3139-9900)	3.654 (2.71-6.98)
P-3: mild infiltration (n = 10)	2.39 (0.89-5.38)	1.78 (0.84-2.0)	0.128 (0.093-0.299)	1.50 (0.87-2)	1.86 (1.05-2.21)	1.56 (0.748-1.85)	1.25 (0.96-1.92)	4453 (2044-6290)	3.89 (2.63-6.42)
P-4: Infiltration with edema (n = 5)	1.86 (0.60-2.46)	1.16 (0.83-1.90)	0.272 (0.101-0.312)	1.0 (1-2)	1.12 (0.955-2.24)	1.60 (0.75-2.29)	1.04 (0.424-2.09)	4086 (3139-18900)	2.85 (2.04-3.64)

MRI, magnetic resonance imaging; ^18^F-FDG SUV_max_, 2-[18F]-fluoro-2-deoxy-D-glucose target tumor maximum standardized uptake value; P-1:P-4, Pattern 1 to 4; CE, contrast enhancing; NE, non-contrast enhancing; rCBV, relative cerebral blood volume; ADC, apparent diffusion coefficient (10^-3^ mm^2^/s); FA, fractional anisotropy; MD, mean diffusivity (10^-3^ mm^2^/s); Cho/Cr, choline/creatine; Cho/NNA, choline/N-acetyl aspartate; NAA/Cr, N-acetyl aspartate/creatine; LL, lipids/lactate.

Correlation values were calculated by measurements from 41 biopsy specimens. They are expressed as median and range values. Seven biopsy specimens from the CE region were excluded because they contained necrosis.

The highest value of LL ratio was found in the CE region because it may contain necrosis in the biopsy specimen.

The Ki-67/MIB-1 LI was significantly associated with the Cho/Cr ratio (*r* = 0.95, *p* = 0.03), whereas a borderline association was found with rCBV values. Furthermore, Ki-67/MIB-1 LI was strongly associated with ^18^F-FDG SUV_max_ in the CE region *(r* = 0.87, *p* = 0.008), also in presence of necrosis (*r* = 0.72, *p* = 0.006).

rT2 FSE and rT2 FLAIR were not specific for edematous areas, whereas rCBV, Cho/Cr and Cho/NAA ratio values were higher and FA values lower in edematous areas with infiltration than without infiltration.

In the CE region, rCBV, Cho/Cr, Cho/NAA and ^18^F-FDG SUV_max_ values provided the best evaluation of the GB malignancy and corresponded to the highest Ki-67/MIB-1 LI. ^18^F-FDG SUV_max_, rCBV, Cho/Cr and Cho/NAA decreased from P-2 to P-4 (Figure 2B−2D). In peritumor regions, ^18^F-FDG SUV_max_, Cho/Cr and Cho/NAA were still capable of detecting tumor infiltration. ADC and FA were not suitable tumor indicators (*p* > 0.05 for both).

### Immunohistochemistry (IHC)

**Figure 3:**
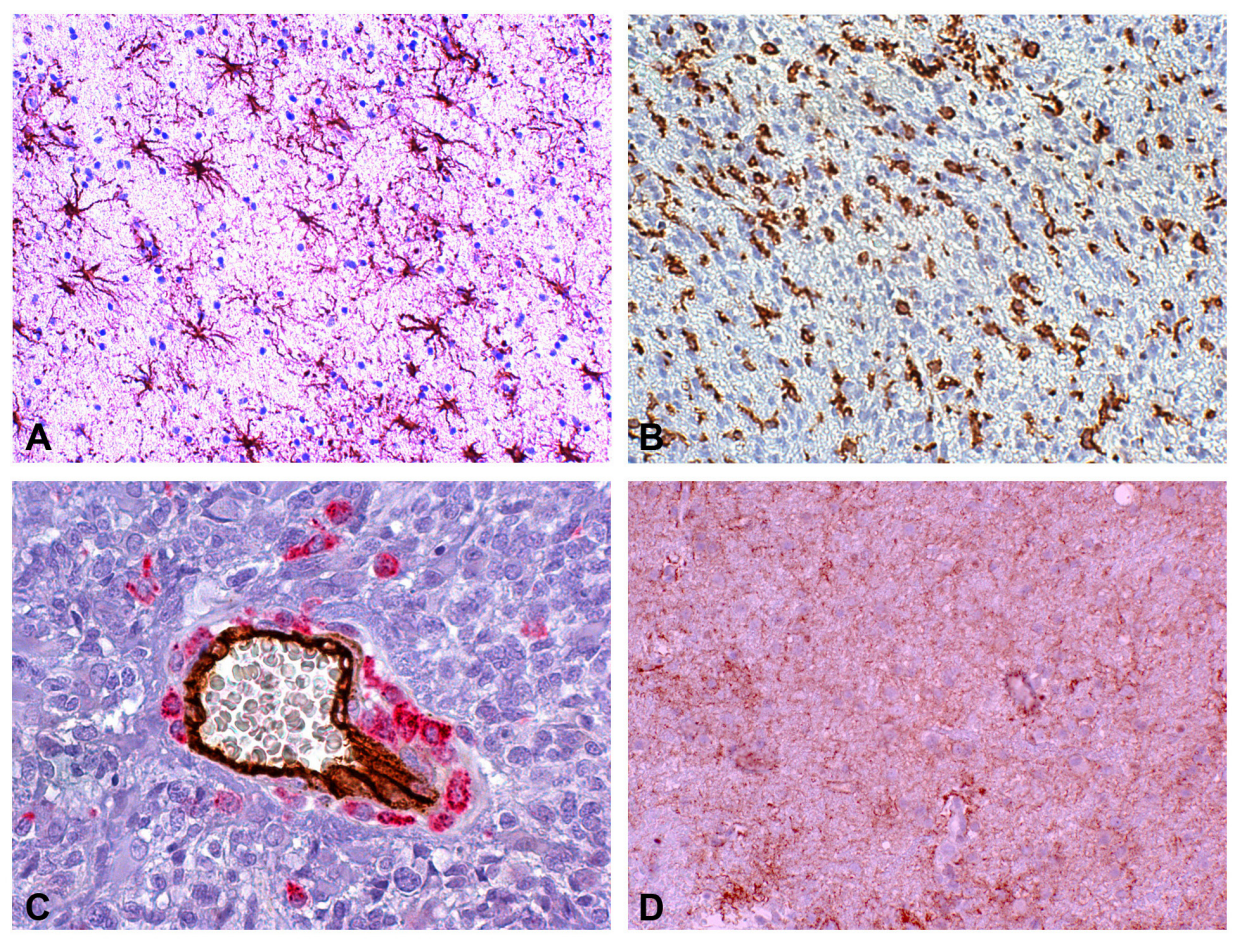
Reactive astrocytes, microglia/macrophages and edema in a peritumor stereotactic biopsy specimen **(A)** Reactive astrocytes at regular interdistance. GFAP (DAB, 200×). **(B)** Reactive ramified microglia and macrophages in an infiltration area. Iba-1 (DAB, 200×). **(C)** Scattered and clustered macrophages around a middle size vessel and isolated macrophages in tumor parenchyma. Double staining CD34-CD68 (DAB and Alkaline Phosphatase Red, respectively, 400×). **(D)** Aquaporin 4 immunopositivity in reactive astrocytes and vessels (DAB, 200×). GB, glioblastoma; GFAP, glial fibrillary acidic protein; Iba-1, ionized calcium-binding adapter molecule 1; DAB,3,3'-Diaminobenzidine.

IHC revealed (i) variable glial fibrillary acidic protein (GFAP)-positive staining of tumor cells and constant and strong staining of reactive astrocytes (Figure 3A), (ii) Iba-1/CD68-positive staining of reactive ramified microglia (Figure 3B) and macrophages (Figure 3C), (iii) CD34-positive staining of endothelial cells (Figure 3C) and IDH1^R132H^-negative immunostaining. ATRX expression was positive in tumor cells and reactive astrocytes. Edematous areas were characterized by intense expression of Aquaporin 4 both on reactive astrocyte processes and vessels (Figure 3D).

### Reactive astrocytes

In P-1 and P-3, GFAP expression and morphology may allow to detect reactive astrocytes. Their number was variable in P-1, whereas in P-2 and P-3 they were easily recognizable with a frequency of 20-25 *per* 400× high-power field (HPF) or more, depending on the supposed time elapsed from the infiltration. In comparison with areas of tumor infiltration, the number of reactive astrocytes slightly decreased if edema was present (Table 3).

**Table 3:** Cell counts in the different tissue areas

Tissue area	Cellularity	GFAP+ reactive astrocytes	CD68 + cells	Iba-1+ cells
Normal cortex	240±20	–	0	10±3
Normal white matter	240±30	–	0	10±3
P-1: CE tumor	450±50	Variable	100±10	70±5
P-2: High infiltration with MVPs	400±40	20±5	2±1	100±10
P-3: Mild infiltration	350±30	25±7	2±1	100±10
P-4: Infiltration with edema	300±40	20±7	0	100±10

CE, contrast enhancing; GFAP, glial fibrillary acidic protein; Iba-1, ionized calcium-binding adapter molecule 1; P-1:P-4, Pattern 1 to 4; MVP, microvascular proliferation.

Cell counts were obtained at 400×HPF. Values are presented as means ± SD.

### Microglia/macrophages

In the CE region, most of the cells showed the morphology of macrophages and intermediate forms from ramified microglia. Occasionally, they could be clustered around vessels or necroses. In areas with infiltration, the aspect corresponded to reactive ramified microglia. Macrophages showed a granular immunopositivity for CD68, while reactive ramified microglia displayed a diffuse immunopositivity for Iba-1. The frequency of macrophages was variable in the biopsy specimen from the CE region (up to > 100 elements *per* 400× HPF); the number of reactive ramified microglia in areas with infiltration was equal to ∼100 elements (*per* 400× HPF). In edematous areas, the number of reactive ramified microglia decreased from < 100 to 5-10 elements (*per* 400× HPF). Microglia cells were frequent in all histological patterns, whereas macrophages were frequent in the CE region and low far from it (Table 3).

### *In vitro* cultures

**Figure 4:**
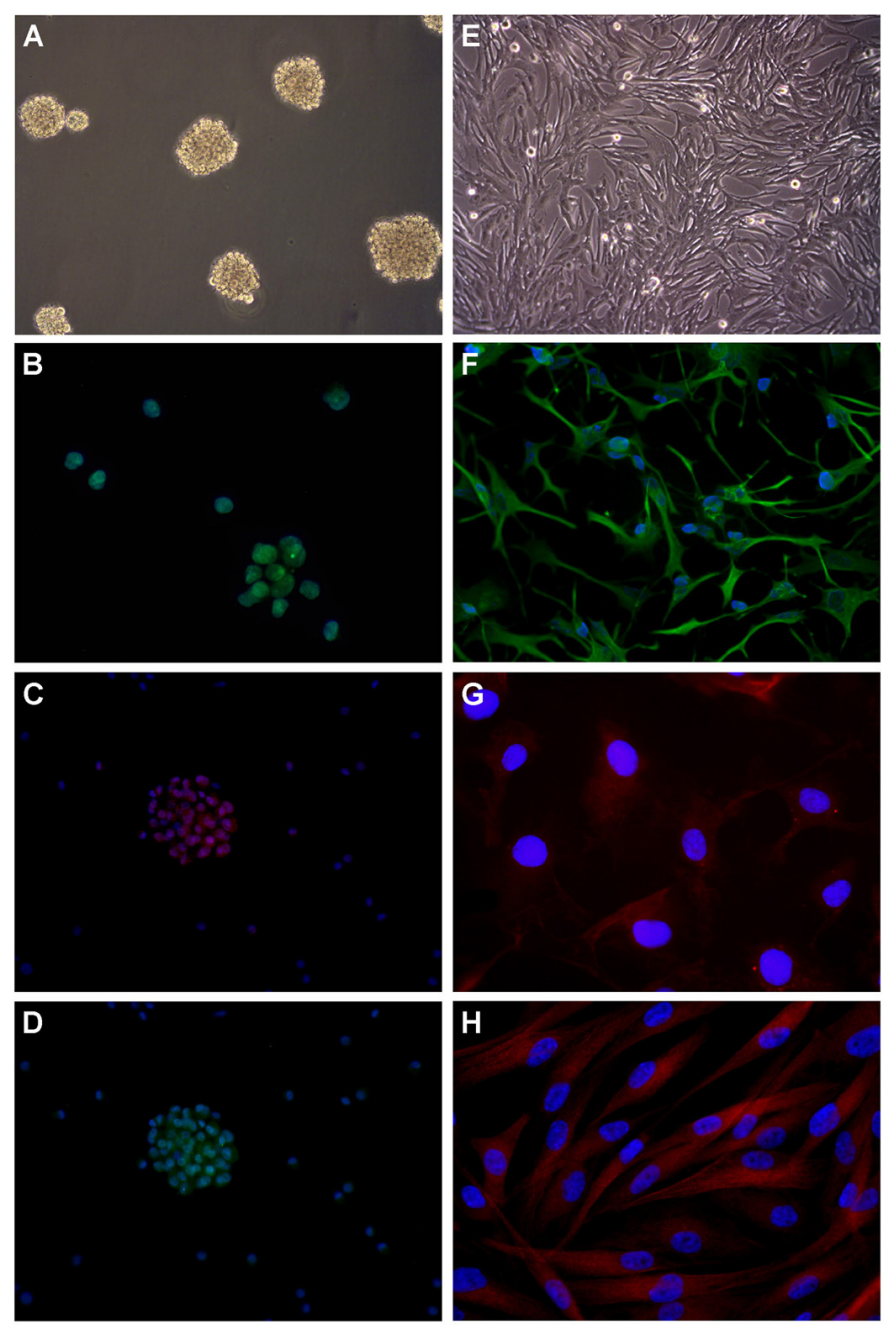
Development of cell lines by *in vitro* culture of newly diagnosed GB and immunofuorescence **(A)** Neurospheres in DMEM/F-12 medium supplemented with growth factors (100× magnification). **(B)** SOX2 expression in nuclei (400× magnification). Nuclei are counterstained with DAPI. **(C)** CD133 expression in cytoplasms (200× magnification). **(D)** Musashi.1 expression in cytoplasms (200× magnification). **(E)** Adherent cells in DMEM supplemented with serum (100× magnification). **(F)** GFAP expression in cytoplasms (200× magnification). **(G)** GalC expression in cytoplasms (400× magnification). **(H)** βIII Tubulin expression in cytoplasms (400× magnification). DMEM, Dulbecco’s modified Eagle’s medium; DAPI, 4’,6-diamidine-2’-phenylindole, dihydrochloride; SOX2, sex determining region Y-box 2; GFAP, glial fibrillary acidic protein; GalC, galactosylceramidase.

The development of neurospheres (NS) or adherent cells (AC) (Figure 4A and 4E) from the different biopsy specimens with respect to MRI and histological patterns is illustrated in Table 4. NS mainly developed in biopsy specimens obtained from the CE region (P-1), and, to a lesser extent, in specimens containing P-2 or P-3. Their development progressively decreased in biopsy specimens obtained from more peripheral NE regions; AC followed a similar pattern.

**Table 4:** Development of neurospheres and adherent cells with respect to MRI and the different histopathological features

Histological pattern/MRI	Neurospheres	Adherent cells
P-1: CE tumor	++	+
P-2: High infiltration with MVPs	+	+
P-3: Mild infiltration	+/–	+
P-4: Infiltration with edema	–	–

MRI, magnetic resonance imaging; P-1:P-4, Pattern 1 to 4; CE, contrast enhancing; MVP, microvascular proliferation.

For neurospheres, the scale refers to their number, size and growth rate. For adherent cells, the scale refers to the presence or absence of a stabilized monolayer.

As revealed by immunofluorescence (IF), NS constantly expressed stemness antigens, such as Nestin, SOX2 and REST, but only sporadically CD133 and Musashi.1 (Figure 4B–4D). AC expressed differentiation antigens, such as GFAP, GalC and βIII-Tubulin (Figure 4F–4H). Stemness antigens have been observed in all biopsy specimens from the CE region as indicators of malignancy.

### Molecular genetics

The genetic and epigenetic alterations identified in the biopsy specimens from the CE region included loss of heterozygosity (LOH) on 10q (9/12, 75%), LOH on 9p (4/12, 33.3%), LOH on 17p (3/12, 25%), PTEN mutations (4/12, 33.3%), TP53 mutations (3/12, 25%), TERT promoter mutations (10/12, 83.3%), EGFR amplification (4/12, 33.3%) and MGMT hypermethylation (7/12, 58.3%). No *IDH1/2* somatic mutations at Arg132 and Arg172 hot spot codons were detected. Most of the genetic and epigenetic alterations were identified in biopsy specimens from the CE region showing the highest values of ^18^F-FDG SUV_max_, rCBV, Cho/Cr ratio and the most malignant tumor phenotype (P-1). They recapitulated the molecular profile identified in the surgically removed tumor. The spectrum of genetic and epigenetic changes overall decreased from CE to NE regions to get undetectable in biopsy specimens from mildly infiltrated NE areas (Table 5). The molecular alterations were therefore identified in the CE region and in highly infiltrated areas (P-1 and P-2).

**Table 5:** Comparison among histological patterns, MRI and molecular genetics

Histological pattern	MRI	LOH^9p,10q,17p^	EGFR^Ampl^	TERT^Mut^	PTEN^Mut^	TP53^Mut^	MGMT^Meth^
P-1: CE tumor	CE	+	++	++	++	+	+
P-2: High infiltration with MVPs	CE/NE	+	+	+	+	+	+
P-3: Mild infiltration	CE/NE	+/–	+	–	–	–	–
P-4: Infiltration with edema	NE	–	–	–	–	–	–

MRI, magnetic resonance imaging; P-1:P-4, Pattern 1 to 4; MVP, microvascular proliferation; CE, contrast enhancing; NE, non-contrast enhancing; LOH, loss of heterozygosity; *EGFR*, epidermal growth factor receptor; *TERT*, telomerase reverse transcriptase; *PTEN*, phosphatase and tensin homologue; *TP53*, tumor protein p53; *MGMT*, O-6-methylguanine-DNA methyltransferase.

LOH: The scale corresponds to the presence (+) or absence (–) of LOH on the tested regions; *EGFR*: The scale corresponds to the number of samples with gene amplification (++, >50%; +, <50%; –, no amplification); *TERT*, *PTEN*, *TP53*: The scale corresponds to the number of samples with gene mutations (++, >50%; +, <50%; –, wild type); *MGMT*: The scale corresponds to the number of samples with (+) or without (–) promoter methylation.

As for GB-derived cell lines, the genotype observed in NS totally mimicked the genotype of the solid tumor; it was absent or only partially expressed in AC. The nucleotide sequence variations identified in *TERT*, *PTEN* and *TP53* genes are listed in the Supplementary Table 1. All of them were of somatic origin.

## DISCUSSION

Conventional and advanced MRI, combined with ^18^F-FDG PET/CT, may reveal the biological significance of the different GB tumor areas when compared to the tumor phenotype and genotype, consistently with previous observations [26, 27], also after frameless image-guided stereotactic neurosurgery [28, 29]. Sensitivity, specificity, positive predictive value (PPV) and negative PV (NPV) of conventional MRI are not satisfactory in predicting glioma grade. Conversely, rCBV and metabolite ratio measurements from MRSI, alone or combined, can significantly improve both sensitivity and PPV of conventional neuroimaging [11]. Significant differences in rCBV measurements and Cho/Cr, Cho/NAA or NAA/Cr ratio have been reported between low and high grade gliomas, with rCBV having the greatest diagnostic performance [11]. In the clinical practice, ^18^F-FDG PET/CT is one of the most widely used tracer to indicate the presence of a tumor. We considered ^18^F-FDG SUV_max_ as a reliable indicator of tumor malignancy if compared to other volume-based PET parameters, such as metabolic tumor volume (MTV) or total lesion glycolysis (TLG). Indeed, MTV needs the definition of an operator-based threshold that can introduce variability. Conversely, the global marker TLG cannot correlate imaging with histopathology of strictly delimited biological specimens as we required. Moreover, in order to increase the spatial resolution, we fused ^18^F-FDG PET/CT with MRI (^18^F-FDG PET/CT/MRI fusion images).

### Is the *in vivo* identification of the most malignant tumor areas achievable?

In our series, imaging of the CE region, corresponding to the histologically most malignant tumor areas, shows higher values of MRI rT1C and rCBV, MRSI Cho/Cr, Cho/NAA, LL ratio and ^18^F-FDG SUV_max_ in comparison with NE regions. rT2 FSE, rT2 FLAIR, ADC and FA do not show clear-cut differences, in agreement with previous findings [6–8]. The reason why rT2 FSE or rT2 FLAIR cannot discriminate among the different regions of the tumor could be due to the heterogeneity of the water content in the ROI. Amid the conventional MRI parameters, rT1C was the most reliable to discriminate high malignancy regions. Remarkably, in all biopsy specimens from the CE region, rCBV values are constantly and significantly increased if compared with the threshold of 1.75 considered as indicative of GB [11]. Relatively lower rCBV values may be explained by necrosis. Additionally, all biopsy specimens from the CE region are characterized by the highest values of Cho/Cr, Cho/NAA and LL ratio. These parameters (except for LL) can decrease in presence of necrosis, even though they remain high compared with specimens from NE areas. The highest values of LL ratio are caused by the occurrence of necrosis in biopsy specimens from the CE region. This is due to the location of circumscribed necroses in hyperproliferative areas as emblem of malignancy. It is quite obvious that the NAA/Cr ratio is higher far from the CE region as well as in areas with infiltration.

Among perfusion parameters, rCBV, PH and PSR measurements correlate with tumor microvasculature, as already observed [6–8]. rCBV variations provide a reliable index of the tumor microvasculature and histological grade [10, 12, 13, 30]. In our series, rCBV values decrease from CE to NE regions with higher values in infiltrated areas with MVPs indicating neoangiogenesis.

In literature and in our study, rCBV is significantly associated with ^18^F-FDG SUV_max_ or to local metabolic rate of glucose on PET [31]. In adult brain tumors, imaging with ^18^F-FDG PET/CT showed that an increased ^18^F-FDG uptake corresponds to an increased tumor malignancy, aggressiveness, grade [21], cell density and rCBV [32]. This has also been observed in pediatric brain tumors [33].

From our data, ^18^F-FDG PET/CT/MRI fusion images indicate the most malignant tumor phenotype, corresponding to the histological P-1, as reported in literature [5, 31]. On the other hand, ^18^F-FDG SUV_max_ is associated with the Ki-67/MIB-1 LI in CE regions, also if containing necrosis, confirming a recent meta-analysis [34]. However, a possibility of mistake in the ^18^F-FDG SUV_max_ interpretation can occur when the ROI includes the cerebral cortex due to physiological neuronal metabolism.

Remarkably, glioma grade can be successfully predicted by the significant association of rCBV, Cho/Cr ratio and ^18^F-FDG SUV_max_ with the histological P-1 and the Ki-67/MIB-1 LI [9].

No correlation has been found between ADC values and the histopathological findings. ADC values of tumor parenchyma can indirectly reflect cell proliferation and malignancy [35, 36]. They should be inversely correlated with tumor grade [9, 14, 15], with low values in hypercellular areas [37]. Even though an inverse correlation between ^18^F-FDGSUV_max_and ADC_min_, and between SUV ratio and ADC_min_ has been reported [38], ADC is not regarded as a suitable parameter for tumor grade identification [16, 17]. We obtained similar ADC values in CE (where the high cellularity should restrict water molecules in their movement) and NE regions, as well as a lower ADC value in edema, where water is abundant. Several factors may affect ADC measurements. For example, an enlargement of the extracellular compartment caused by necrosis could likely cancel the effect of high cellularity. On the other hand, also the presence of non-tumor reactive cells (*i.e.* astrocytes and microglia/macrophages) in edematous areas could reduce ADC.

Reduced FA values are observed in both CE and NE regions if compared with normal brain. The reduction is greater in infiltrated areas compared with the CE region without necrosis or to areas of high infiltration with MPVs, where relatively higher FA values reflect the presence of microstructural barriers related to the high cell density and vascular proliferation. The reduction of FA caused by necrosis may explain the variability of the measurements and the contrasting results from the literature when FA has been used as index for tumor grade [9, 15, 36, 39, 40]. From our study, FA does not result a suitable parameter to assess tumor malignancy.

The relationship of ^18^F-FDG SUV_max_ and MRI parameters with molecular features of GB is not direct but mediated by histology. In general, neuroimaging corresponds to histology and histology, in turn, to molecular genetics. Previous attempts to correlate neuroimaging with molecular genetics have been made. For instance, a significant correlation has been found between rCBV [41] or ADC values [42] and *EGFR* gene amplification/*EGFR*vIII. However, only 40% of GBs harbors *EGFR* gene amplification/*EGFR*vIII [43]. Likewise, the correlation of ADC values with the *MGMT* hypermethylation can be affected by the unreliability of ADC in GB [44].

The detection of *IDH1/2* mutations through the measurement of the 2-hydroxyglutarate (2-HG) oncometabolite levels by MRSI, despite a possible spectral overlap with other metabolites, would be remarkable only if referred to the whole glioma group [37]. In our series, all cases are *IDH*-wild type GBs.

Interestingly, different genetic expression patterns between CE and NE regions have been reported by gene microarray studies, with genes associated with mitosis, angiogenesis and apoptosis clustering in the former [6]. In our series, allelic imbalances on the critical chromosomal regions 9p, 10q and 17p, *EGFR* gene amplification, *TERT*, *PTEN* and *TP53* mutations, and *MGMT* hypermethylation prevail in CE compared with NE biopsy specimens. However, their full expression is only detectable in the CE region and in highly infiltrated areas. The number of genetic and epigenetic alterations progressively decreases from the most malignant tumor regions to peripheral ones, parallel to the regional variations of ^18^F-FDG SUV_max_ and MRI parameters. The molecular heterogeneity can be due, beside policlonality, to the rarefaction of tumor cells moving away from the proliferative tumor [45, 46]. In order to correlate regional genetic heterogeneity and spatially matched imaging measurements, some authors recently analyzed DNA copy number variants for core GB driver genes, as reported by The Cancer Genome Atlas, on multiple image-guided biopsies in primary GB patients from CE and NE regions [47]. Interestingly, neuroimaging displayed a significant correlation with 6 drivers genes (*EGFR*, *PDGFRA*, *PTEN*, *CDKN2A*, *RB1*, *TP53*) supporting, in agreement with our findings, radiogenomics in characterizing regional genetic heterogeneity in GB.

### Is the extent of tumor infiltration *in vivo* detectable?

The invasion capacity of tumor cells contributes to the failure of a local control of the tumor, because infiltrating tumor cells can extend far from it escaping radiation effects. They are detected in peritumor edema [48] and in normally looking areas in 20% of GBs [49]. MRI fails to detect them when occurring inT2 hyperintense or normal T1 or T2 areas [50]. The recognition of tumor infiltration in edematous areas is obviously important, although it has been observed that the removal of T2 hyperintense NE areas does not impact on patient outcome [51].

In MRI edematous areas, histologically characterized by an increased expression of Aquaporin 4 [52], the identification of tumor cells depends on their frequency. Indeed, edema influences both MRI variables and the total cell number, regardless of their nature (tumor, normal, endothelial cells, microglia/macrophages or inflammatory cells). The bias is represented by the difficulty to recognize tumor infiltration when it overlaps with edema [53, 54]. In edematous areas, tumor and normal cell density decreases and tumor infiltration is only detectable when tumor cell density is higher than normal, at least higher enough to reach the threshold influencing MRI variables [25, 55].

GB frequently spreads along white matter tracts causing disruption that can be detected by DTI. However, no significant differences in FA measurements have been found in infiltration areas with or without MVPs and with edema. This is in agreement with previous studies showing a low sensitivity of FA reduction to recognize infiltration [56] or doubts on its reliability [57, 58].

A high Cho/NAA ratio in edematous areas allows detecting tumor infiltration [59–61], while low absolute values of total NAA seem to be more effective than Cho to reveal low tumor infiltration [62]. In our series, ^18^F-FDG SUV_max_, Cho/Cr and Cho/NAA ratio, result the most suitable parameters to detect tumor infiltration in edematous areas.

Interestingly, reactive astrocytes, typically distributed at regular intervals, never exceed a constant number *per* field (20-25 at 400× HPF). Their number can influence the cell counts of an area and, consequently, neuroimaging only when it contains < 100 tumor cells *per* field. Microglia/macrophages, which exceed four times the number of reactive astrocytes, could mimic tumor cell infiltration. Tumor cell infiltration beneath a certain value is undetectable. Therefore, the detection of ITCs in peritumor areas or, even worse, far from the tumor, remains unachievable thus representing a sword of Damocles for tumor recurrence.

### Is the *in vivo* identification of the tumor sites responsible for the GSC origin possible?

Therapies for high grade gliomas are ineffective when addressed to the insensitive tumor mass; they should be targeted to the cells responsible for the growth, *i.e.* GSCs. These are believed to occur everywhere in the tumor [63] or to be heterogeneously distributed within it [64, 65]. Regardless of their origin [25, 55, 66], our findings demonstrate a molecular and stemness gradient from the highest malignant tumor areas to the periphery [67, 68].

The only methods for the *in vivo* detection of GSCs are either the neurosphere assay (NSA) [69] or the side population assay [70] applied to surgical tumor specimens. Neuroimaging techniques have been successfully used to *in vivo* detect hematopoietic and leukemic stem cells, but their application to solid tumors was limited [45]. Using intravital microscopy, tumor propagation of labeled GSCs has been obtained by direct *in vivo* evidence [45]. Bone marrow-derived endothelial precursor cells, labeled with superparamagnetic iron oxide nanoparticles, have been observed in glioma-bearing severe combined immunodeficient (SCID) mice by MRI [71], but no similar procedure has been adopted for GSCs. The only way to detect the most malignant tumor areas containing GSCs is the combined use of MRI and 18F-FDG PET/CT [55, 72].

Maybe, the search for GSCs as a cell type is misleading. GSCs could represent a functional status [73, 74] in dynamic equilibrium with tumor non-stem cells under the regulation of microenvironment [75–77]. Our current findings suggest that GSCs are generated or reside in the most malignant tumor phenotype [65].

This study has two limitations: (i) the relatively small sample size, responsible for the low number of comparisons, and (ii) possible technical pitfalls derived from a misregistration between the biopsy site and the region of interest (ROI) imported into the neuronavigation device (although all stereotactic biopsies have been performed before craniotomy).

## MATERIALS AND METHODS

### Patients

In an initial database we identified 23 consecutive potentially eligible patients from 2012 to 2014 at the Department of Neurosurgery of Città della Salute e della Scienza (Turin, Italy). Among these, twelve patients (7 men, 5 women; mean age 65 y, 48 – 78) (labeled CTO3, CTO4, CTO5, CTO6, CTO10, CTO11, CTO12, CTO14, CTO15, CTO16, CTO17, CTO23) were then selected according to the eligibility criteria [i.e. newly diagnosed GB (79)] and consent to participate in the study.

The study was in compliance with the local institutional review board and Committee on Human Research and with the ethical human-subject standards of the World Medical Association Declaration of Helsinki Research. Written informed consent was obtained from all patients.

### Preoperative MRI

Preoperative MRI was performed one or two days before the planned biopsy on a 1.5-tesla (1.5T) MR scanner (GE Medical Systems, Milwaukee, WI, USA). The optimized scan protocol included both conventional and advanced (DWI, DTI, PWI and MRSI) MR techniques.

Conventional MRI included sagittal and axial T1-weighted spin echo (SE) images (TR/TE = 560 ms/14 ms), axial Proton Density-T2-weighted fast SE (PD-T2 FSE) (TR/TE = 3480 ms/24.2-105 ms), coronal T2-weighted fast SE (T2-FSE) (TR/TE = 8040 ms/118 ms) and sagittal isotropic 3-dimensional fluid attenuated inversion recovery (3D-T2 FLAIR) (TR/TE/inversion time = 6000 ms/133 ms/2000 ms). Contrast enhanced T1-weighted SE images and 3D Spoiled Gradient Recalled T1-weighted SE (3D SPGR T1 TSE) (TR/TE = 10.54 ms/4.20 ms; 1.6 mm/0 mm, slice thickness/interslice gap).

DWI data were acquired using single shot, SE, echo-planar imaging (EPI) (TR/TE = 6100 ms/98.6 ms; 5 mm/1 mm slice thickness/interslice gap; 128 × 128 matrix). DWI datasets consisted of 3 independent gradient directions at *b* = 1000 s/mm^2^ and 1 non-diffusion weighted image (b = 0 s/mm^2^).

DTI data were acquired using single shot EPI-SE (TR/TE = 12000 ms/∼96.7 ms) with 2.5 mm isotropic voxels. DTI datasets consisted of 48 imaging volumes: 25 independent gradient directions at b = 1000 s/mm^2^ and b = 0 s/mm^2^.

Axial dynamic susceptibility contrast (DSC)-PWI was performed using a series of T2* EPI-FID (flip angle = 90°; TR/TE=1725 ms/50 ms; parallel imaging asset with acceleration factor 2; 128 × 128 matrix; 28 × 28 cm field of view; 20 slices, 5-mm slice thickness, 35 time points) before, during and after intravenous administration of 0.1 mmol *per* kilogram body weight of Gadobutrol (Gadovist^®^, Bayer HealthCare Pharmaceuticals, Berlin, Germany). The first 6 EPI acquisitions were performed before the injection of Gadobutrol to establish a pre-contrast baseline. Pre-injection of 2 mL of contrast material was administered in order to reduce the influence of T1. The region selected for DSC coverage included the entire supra-tentorial area.

3D H-1 MRSI (TR/TE = 1000 ms/144 ms with 1-cc nominal resolution) was acquired before contrast administration to cover the entire tumor volume as determined by T2-weighted FLAIR and FSE images together with the normal appearing adjacent brain tissue.

### Advanced MRI – image post-processing

Advanced MRI data were analyzed using a commercial post-processing software (FuncTool, v3.1, GE Medical Systems). For diffusion analysis, ADC maps were created and for perfusion analysis, color-coded CBV maps were generated after motion correction to provide better visualization of tumor boundaries. CBV was calculated as the area under the curve of the first-pass bolus.

The spectra were automatically phased, frequency aligned and baseline corrected, and spectral parameters were calculated. Cho, Cr, NAA and LL peak areas were estimated integrating the resonances at 3.2, 3.0, 2.0 and 0.6-1 ppm, respectively. Normal values for each metabolite peak area were determined by averaging values in 10 to 20 voxels selected within brain parenchyma with a normal MR appearance, usually in the contralateral hemisphere to the tumor.

For each T1 brain scan, ROIs (5-mm diameter circumferences) were drawn and individual masks for each brain were generated. The ROI size was intermediate between PET/CT and MRI spatial resolutions.

The initial processing step was the Brain Extraction Toolkit (BET) (FSL, www.fmrib.ox.ac.uk/fsl) to isolate the brain from the background. DTI acquisition was corrected for eddy currents and head-motion distortions (using the FSL program Eddy Correct) and FA maps were obtained with the Frequent Domain Transform Toolbox in FSL. FA and MD images of all subjects were aligned to the respective T1 by a non-linear registration (FNIRT, www.fmrib.ox.ac.uk/fsl); transformation matrices were then generated and applied to ROIs. ROI value analysis was performed by ImageJ software (http://imagej.nih.gov/ij/).

Contrast enhanced 3D SPGR T1-weighted images were fused with ^18^F-FDG PET/CT images using the Integrated Registration software on the Advantage image processing workstation (GE Medical Systems) to further increase spatial resolution.

### ^18^F-FDG PET/CT

Patients fasted for at least 6 hours prior to examination. Before radiopharmaceutical injection, blood glucose was measured (< 130 mg/dL in all cases). Each subject was intravenously injected with approximately 185 MBq of ^18^F-FDG 60 minutes before the scan. ^18^F-FDG PET/CT scans were then acquired by a Discovery ST-E PET/CT System (GE Medical Systems) combining a helical multislice CT scanner and a designed Bismuth germanate (BGO) block detector PET tomograph, in 3D modality with a total axial field of view of 30 cm and no interplane gap space. ^18^F-FDG PET/CT images were acquired through 2 sequential scans: CT brain scan (thickness, 3.75 mm; 140 kV; 60-80 mA/s) and PET brain scan [Field of View (FOV) of 30 cm, transaxial]. The PET scan was initiated immediately after the CT examination in order to use CT data for the attenuation correction of the PET data. 3D data were reconstructed using a 3D-OSEM algorithm (VUE-point) with the corrections (random, scatter, attenuation) incorporated into the iterative process. The parameters were: number of subsets 28; number of iteration 4. Data were collected in 128 × 128 matrices with a reconstructed voxel of 1.33 x 1.33 x 2.00 mm. Finally, ^18^F-FDG PET/CT images were fused with contrast enhanced 3D SPGR T1-weighted images using the Integrated Registration software on the Advantage image processing workstation (GE Medical Systems).

### Preoperative tumor tissue site selection, MRI and ^18^F-FDG PET/CT analysis

ROIs were defined on the contrast enhanced 3D SPGR T1-weighted MR sequence from the CE region of the tumor to progressively more peripheral NE regions (< 1 cm and >1 cm from the CE region). The exact location of the ROI was the best achievable compromise between the minimal neurosurgical risks (*i.e.* intracranial bleeding) and the representative chosen tumor areas. The sampling sites varied in number from 3 to 5: at least 1 of these was selected in the CE region of the tumor and 2 in progressively more peripheral NE regions < 1 cm and >1 cm from the enhancing ring, in peritumor edema and normal tissue. The T1C, T2 FSE, T2 FLAIR and CBV values were normalized dividing the estimated values in the normal-appearing white matter from the contralateral hemisphere and expressed as relative values (r). Metabolite concentrations of Cho, Cr, NAA were obtained and their ratios calculated. Finally, the ^18^F-FDG SUV_max_was calculated on the ROIs identified by PET/CT/MRI fusion images. Furthermore, SUV_max_ of white matter homolateral and contralateral to the lesion, SUV_max_ of grey matter homolateral and contralateral to the lesion, SUV_max_ of ventricle were calculated in order to obtain the ratio SUV_max_-lesion/SUV_max_-white matter homolateral and contralateral to the lesion, SUV_max_-lesion/SUV_max_-grey matter homolateral and contralateral to the lesion and SUV_max_-lesion/SUV_max_-ventricle.

Co-registration of ^18^F-FDG PET/CT and advanced MRI data was achieved. ROIs defined on the PET/CT/MRI fusion images were copied to the co-registered MRI dataset and used to calculate the average metabolite concentrations for Cho, Cr, NAA and Cho/NAA ratio in the corresponding areas. Congruence of areas for ^18^F-FDG uptake and the above mentioned metabolic ratio in the lesions was estimated on the co-registered ^18^F-FDG PET/CT and MRI datasets.

Biopsy sampling sites were reviewed together with the neurosurgeon in order to establish the feasibility of the biopsy

### Stereotactic brain biopsy

All 12 patients underwent frameless MRI-guided stereotactic brain biopsy by the same neurosurgeon. General anesthesia was induced and patient’s head fixed in a three point Mayfield clamp in which was attached a Vertek^®^ II Articulating Arm with the neuronavigation reference star (Small Passive Cranial Reference Frame) (Medtronic Inc., Louisville, CO, USA). Forehead and facial surface anatomical landmarks were obtained with the Tracer registration method.

For each patient, the preoperative CE 3D SPGR T1-weighted MR images were imported into the Stealth Station^®^ Treon Plus™ Surgical Navigation System (Medtronic) in order to identify all biopsy targets and to establish stereotactic coordinates. Intraoperative image guidance was achieved using the Mach™ Cranial Planning 4.6. software (Medtronic).

Biopsies were performed through a single 14-mm diameter burr hole at the defined stereotactic sites using a Navigus^®^ Frameless Biopsy System including the Biopsy Trajectory Guide Kit and a Passive Biopsy Needle (Medtronic). For each patient, from 3 to 5 biopsy specimens, approximately 8-mm-long and 1-mm in diameter, were obtained. No bleeding occurred as a complication during the procedure.

Craniotomy, dura opening and full or partial tumor removal were performed after biopsies.

### Pathology and IHC

Each biopsy specimen was split in three fragments. The first one was formalin fixed, paraffin embedded (FFPE) and cut in 5 μm-thick sections. The second one was minced and put in culture and the third fragment was stabilized in RNAlater^®^ solution (Life Technologies, Foster City, CA, USA) and stored at -80°C.

All biopsy specimens and the removed solid tumor of each patient underwent histological diagnosis according to the routine practice.

Beside hematoxylin and eosin (H&E) staining, IHC was performed with the primary antibodies listed in Table 6 on a Ventana Full BenchMark^®^ XT automated immunostainer (Ventana Medical Systems, Tucson, AZ, USA). The Ultra-View™ Universal DAB and Alkaline Phosphatase Red Detection Kits were the detection system (Ventana). Heat-induced epitope retrieval was obtained with Tris–EDTA, pH 8 (Ventana).

**Table 6:** List of primary antibodies used for immunohistochemistry and immunofluorescence

Antibody	Source	Dilution	Code	Company
Ki-67/MIB-1*	Mouse	1: 100	M7240	Dako
GFAP	Mouse	1: 200	M0761	Dako
GFAP °	Rabbit	1: 200	Z0334	Dako
IDH1^RI32H^*	Mouse	1: 20	DIA H09	Dianova
ATRX*	Rabbit	1: 400	HPA001906	Sigma-Aldrich
CD34*	Mouse	Pre-diluted	790-2927	Ventana
CD68*	Mouse	Pre-diluted	790-2931	Ventana
Aquaporin 4*	Rabbit	1: 200	ABI2218	Millipore
Iba-1*	Rabbit	1: 500	#019-19741	Wako
CD16	Rabbit	Pre-diluted	760-4863	Ventana
Nestin*,°	Rabbit	1 : 200	AB5922	Millipore
CD133 °/1 (AC133)	Mouse	1 : 20	130-090-422	Miltenyi Biotec
SOX2*,°	Mouse	1 : 100	MAB2018	R&D Systems
Musashi.1*,°	Rabbit	1 : 200	AB5977	Millipore
REST*,°	Rabbit	1 : 150	IHC-00141	Bethyl Lab.
GalC*,°	Mouse	1 : 100	MAB342	Chemicon
βIII-Tubulin*,°	Mouse	1 : 250	MAB1637	Chemicon

GFAP, glial fibrillary acidic protein; IDH1, isocitrate dehydrogenase 1; ATRX, alpha-thalassemia/mental retardation syndrome X-linked; Iba-1, ionized calcium-binding adapter molecule 1; SOX2, sex determining region Y-box 2; REST, RE1-silencing transcription factor; GalC, galactosylceramidase.

*HIER required, ° used for immunofluorescence.

The following histopathological parameters were evaluated and recorded: cell and vessel density, nuclear atypia, endothelial hyperplasia, MVPs, glomeruli, circumscribed necrosis, mitoses, microglia/macrophages and reactive astrocytes.

Cell density was quantified on H&E-stained sections as the average number of cells manually counted in 5 random microscopic fields, *per* 1000× magnification. Ki-67/MIB-1 LI was calculated as the average percentage of positive nuclei on the total number of nuclei in 5 microscopic fields, *per* 1000× magnification, after visual analysis. Vessel density was estimated on CD34-stained sections as the average number of vessel structures in at least 5 microscopic fields, *at* 400× magnification, using a square grid.

Microglia/macrophages and reactive astrocytes were quantified by manual cell counts as the average number of positive cells on Iba-1-, CD68- and GFAP-stained sections, respectively, in 5 microscopic fields, *per* 400× magnification.

All the above mentioned qualitative and quantitative assessments were performed on each biopsy specimen and on the removed solid tumor.

### *In vitro* cultures

Fresh surgical tissue from each biopsy specimen was processed for *in vitro* culture as previously described [67]. Culture conditions were: Dulbecco’s modified Eagle’s medium (DMEM)/F-12 supplemented with 20 ng/mL epidermal growth factor (EGF) and 10 ng/mL basic fibroblast growth factor (bFGF) for NS development and DMEM supplemented with 10% fetal bovine serum (FBS) for AC development. Cell cultures were maintained at 37°C in a 5% O_2_ and 5% CO_2_ humidified atmosphere. The NS ability to self-renewal, multipotency, clonogenicity and tumorigenicity was assessed as in literature [67].

Cell line authentication from the respective solid tumor was obtained by Short Tandem Repeat (STR) profiling.

Experiments on cell lines were carried out with cells from passages 10−20. Cell lines were monthly checked for *Mycoplasma* contamination before the experimental use (e-Myco™ Mycoplasma PCR Detection kit, iNtRON Biotechnology, Korea).

### IF

IF on cell lines was performed with the primary antibodies listed in Table 6. The secondary antibodies used were Alexa Fluor^®^488-conjugated AffiniPure goat anti-rabbit IgG and Alexa Fluor^®^ 594-conjugated AffiniPure rabbit anti-mouse IgG (Jackson Immuno Research Laboratories Inc., West Grove, PA, USA). Cell nuclei were counterstained with 4’,6-diamidine-2’-phenylindole, dihydrochloride (DAPI). Images were obtained on a Zeiss Axioskop fluorescence microscope (Carl Zeiss, Oberkochen, Germany) equipped with an AxioCam MRc 5 digital camera and coupled to an imaging system (AxioVision Release 4.5, Zeiss).

### Molecular genetics

Genomic DNA (gDNA) from frozen biopsy specimens and cell lines was isolated using the QIAamp DNA Mini kit (Qiagen Inc., Valencia, CA, USA), according to the manufacturer’s instructions. Constitutive gDNA was isolated from peripheral blood by a salting-out procedure.

Molecular genetics analysis was performed on the removed tumor, on each biopsy specimen and on relevant cell lines. Technical procedures were previously published [67, 80, 81].

Allelic imbalances in selected regions (9p, 10q and 17p) were determined by LOH analyses of highly polymorphic microsatellite markers [67]. Fragment analysis and capillary electrophoresis (CE) were performed on an ABI^®^ 3130 Genetic Analyzer (Life Technologies). Data were collected by Gene Mapper v4.0 software (Life Technologies).

The *EGFR* gene amplification status (GenBank accession no. NM_005228) was assessed as previously reported [67].

Methylation specific-Polymerase Chain Reaction (MS-PCR) followed by frament analysis etc and CE was used to determine the *MGMT* promoter hypermethylation status (GenBank accession no. NM_002412). Primer sequences for MS-PCR and amplification conditions were previously reported [80].

*TERT* (minimal promoter region) (GenBank accession no. NM_198253), *PTEN* (exons 1 to 9) (GenBank accession no. NM_000314), *TP53* (exons 2 to 11) (GenBank accession no. NM_000546), *IDH1* R132 (exon 4) (GenBank accession no. NM_005896) and *IDH2* R172 (exon 4) (GenBank accession no. NM_002168) hot-spot codons were amplified by PCR and searched for mutations by Sanger direct sequencing, as published [67, 81]. Cycle sequencing was performed using the BigDye^®^ Terminator v1.1 Cycle Sequencing Kit (Life Technologies). The analysis software was Sequencing Analysis v5.3.1 (Life Technologies). The reported nucleotide and amino acid numbering is relative to the transcription start site (+1) corresponding to the A of the ATG on the GenBank reference sequences and according to the HUGO Gene Nomenclature.

### Statistical methods

Data were checked for Normality by skewness and kurtosis tests. The variations of each neuroimaging parameter were measured determining the median and the range of values observed for each biopsy specimen.

In order to investigate the correlation among the values of MRI and ^18^F-FDG PET/CT parameters, a linear mixed-effects model was used. Beta represents a measure of how strongly each predictor variable influences the criterion (dependent) variable.

Further, two hierarchical linear models were applied to verify the relationship between the Ki-67/MIB-1 LI and the MRI parameters and to assess the relationship between the intensity of ^18^F-FDG PET/CT signal and the distance from the CE region.

The level of significance was set at *p* ≤ 0.05. Statistical analysis was performed using the STATA 13.1 software (StataCorp. LP, College Station, TX, USA).

## CONCLUSIONS

The combined use of conventional and advanced MRI with ^18^F-FDG PET/CT allows identifying different areas of malignant gliomas and to confer them a biological significance. Some parameters (rCBV, Cho/Cr, Cho/NAA and ^18^F-FDG SUV_max_) have a genetic and histopathological correspondence, at least in the CE region. It is then possible to recognize the most malignant areas of the tumor and to detect neoangiogenesis with reliability. However, mild infiltration, especially in edematous tissue, still remains of difficult detection although ^18^F-FDG SUV_max_, Cho/Cr and Cho/NAA ratio are the most suitable parameters to be considered.

The study of tumor heterogeneity on multiple specimens using a frameless image-guided stereotactic navigation system provides very useful information in spite of still persisting technical pitfalls. A prospective observational clinical study (FRONTIER study) has been recently planned to determine the diagnostic accuracy of combined MRI and PET on diffuse gliomas by serial image-guided biopsies in relation to their histopathological and molecular features [78].

## SUPPLEMENTARY MATERIALS TABLE



## Abbreviations

GB: glioblastoma
MRI: magnetic resonance imaging
MRSI: magnetic resonance spectroscopic imaging
^18^F-FDG SUV_max_ PET/CT: 2-[18F]-fluoro-2-deoxy-D-glucose maximum standardized uptake value positron emission tomography/computed tomography
CE: contrast enhanced
NE: non-contrast enhanced
rCBV: relative cerebral blood volume
Cho/Cr: Choline/Creatine ratio
Cho/NAA: Choline/N-acetylaspartate ratio
LL: Lipids/Lactate ratio
ADC: apparent diffusion coefficient
FA: fractional anisotropy
GSCs: glioblastoma stem cells
