# Supplementary material for: Comparison among conventional and advanced MRI, 18F-FDG PET/CT, phenotype and genotype in glioblastoma
Source: Oncotarget. 2017 Oct 4;8(53):91636–53. doi: 10.18632/oncotarget.21482 (PMC5710953; doi:10.18632/oncotarget.21482)
Supplement: Supplementary file 1 [file oncotarget-08-91636-s001.pdf]

Comparison among conventional and advanced MRI, ^{18}F-FDG PET/CT, phenotype and genotype in glioblastoma

SUPPLEMENTARY MATERIALS

Supplementary Table 1: Somatic point mutations in *TERT, TP53* and *PTEN* genes in primary tumors and cell lines

Case	TERT nucleotide change	TP53 nucleotide change	Amino acid change	COSMIC ID.	PTEN nucleotide change	Amino acid change	COSMIC ID.
CTO3	C228T	c.824G>A	p.Cys275Tyr	10893	c.821G>A	p.Trp274*	5162
CTO5	C250T	–	–		–	–	
CTO6	C250T	–	–		c.218A>C	p.Glu73Ala	Not found
CTO10	C250T	–	–		–	–	
CTO11	C250T	–	–				
CTO12	C228T	IVS5-5C>A		Not found	–	–	
CTO14	C250T	–	–		–	–	
CTO15	C250T	c.659A>G	p.Tyr165Cys	10758	c.467G>A	p.Gly156Glu	Not found
CTO16	C228T	–	–		–	–	
CTO17	Wild type	–	–		c.494G>A	p.Gly165Glu	5114
CTO23	C228T	–	–		–	–	

TERT, telomerase reverse transcriptase; *TP53*, tumor protein p53; *PTEN*, phosphatase and tensin homologue; COSMIC, the Catalogue of Somatic Mutations in Cancer (COSMIC v78, released 05-SEP-16, http://cancer.sanger.ac.uk/).
